# Silica- and Silicon-Based Nanostructures

**DOI:** 10.3390/nano12081270

**Published:** 2022-04-08

**Authors:** Céline Ternon

**Affiliations:** Univ. Grenoble Alpes, CNRS, Grenoble INP (Institute of Engineering Univ. Grenoble Alpes), LMGP, F-38000 Grenoble, France; celine.ternon@grenoble-inp.fr

As depicted in Figure 1, studies on silicon and silica-based nanostructures first appeared in the early 1990s, and their numbers grew until the mid-2010s. Since then, the level of scientific research has decreased for silica-based nanostructures and started to decrease for silicon-based nanostructures. An extensive literature review of silicon-based nanostructures clearly shows that the major areas affected by the decline in the number of studies are “applied physics” and “engineering electrical electronic”, whereas numerous applied fields are the subject of increased interest, particularly the applications of energy.

For silicon nanostructures, such a decrease in research is a sign that structures and technologies have been mastered and that the most popular applications, such as electronic devices, have been explored. Research teams are now exploring new areas and applications for these nanostructures.

The purpose of this Special Issue is to bring together state-of-the-art innovations in the field and allow the emergence of novel ideas and concepts for silicon- and silica-based nanostructures. The three reviews in this Special Issue [1,2,3] offer an original view of the last 10 years of research on silicon nanostructures, especially for black silicon [1], bottom-up nanowires [2], and mesoporous silicon [3]. Most interestingly, these reviews outline the areas of interest for the future of these materials. In line with this, some studies [4,5,6,7,8,9,10] clearly illustrate the thematic shift of research into these materials, with a growing interest in eco-responsive areas, such as biomass synthesis [4], environmental sensors [5], batteries [6,7] or depollution [3].

In conclusion, we hope that the readers will enjoy the works and articles collated in this Special Issue, inspiring ideas and providing information to inform further studies of these fascinating nanomaterials, as well as generating new projects and topics.

## Figures and Tables

**Figure 1:**
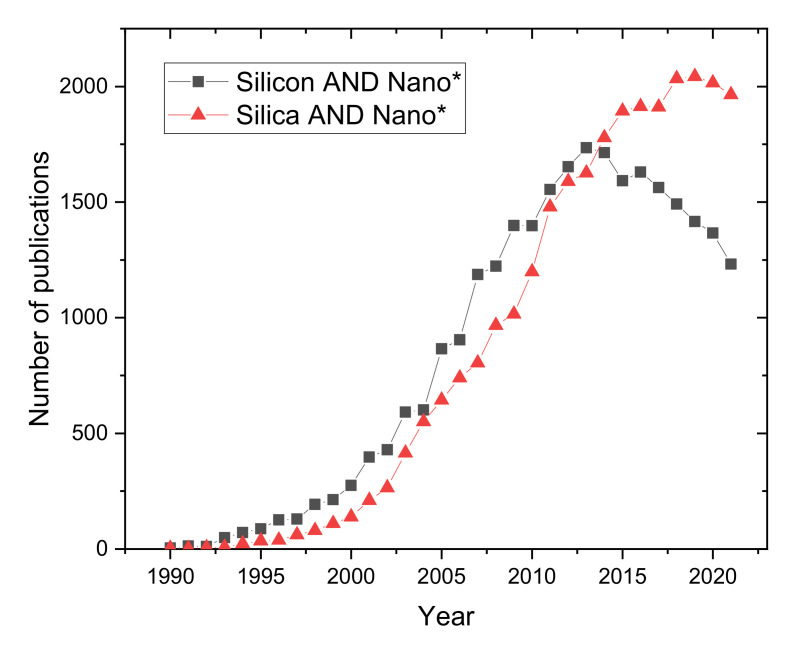
Number of articles published per year about silicon or silica nanostructures, based on a search using the keywords “Silicon AND Nano*” or “Silica AND Nano*” on the website Web of Science. The star * at the end of nano means that all words beginning with “nano” are considered in the search (nanoparticles, nanowires…).
